# Relationship Between Recurrent Pregnancy Loss With Unknown Etiology and Endoplasmic Reticulum Stress

**DOI:** 10.7759/cureus.60899

**Published:** 2024-05-23

**Authors:** Nura F Topbas Selcuki, Pinar Yalcin Bahat, Necip Deniz, Cihan Kaya, Kubra Bagci, Engin Oral

**Affiliations:** 1 Obstetrics and Gynecology, University of Health Sciences, Istanbul Sisli Hamidiye Etfal Training and Research Hospital, Istanbul, TUR; 2 Obstetrics and Gynecology, Kanuni Sultan Süleyman Training and Research Hospital, Istanbul, TUR; 3 Obstetrics and Gynecology, Sanko University Hospital, Gaziantep, TUR; 4 Obstetrics and Gynecology, Bakırköy Dr. Sadi Konuk Training and Research Hospital, Istanbul, TUR; 5 Obstetrics and Gynecology, Yeni Yüzyıl University Gaziosmanpaşa Hospital, Istanbul, TUR; 6 Obstetrics and Gynecology, Biruni University, Istanbul, TUR

**Keywords:** obstetrics, x-box binding protein -1, endoplasmic reticulum stress, oxidative stress, recurrent pregnancy loss

## Abstract

Introduction: Recurrent pregnancy loss (RPL) is characterized by consecutive pregnancy losses before 20 weeks of gestation, with evolving definitions necessitating adjustments to prevent delays in couples' evaluation. Limited etiological data on RPL prompts comprehensive evaluations, often yielding no pathological findings. Emerging research implicates endoplasmic reticulum (ER) stress in various reproductive processes, yet its association with RPL remains understudied.

Aim: To evaluate ER stress in patients with RPL with unknown etiology by determining the plasma concentration of X-box binding protein-1 (XBP-1).

Materials and methods: A total of 45 patients aged 18 to 35 years with at least two pregnancy losses with unknown etiology before the completion of 20 weeks of gestation between March 2020 and September 2020 were included in the study group. The control group consisted of 45 healthy women with at least two previous live births, no pregnancy-associated complications, and no history of pregnancy loss or infertility. The XBP-1 levels were determined from serum samples. Statistical analyses assessed differences between groups, and receiver operating characteristic (ROC) curve analysis determined XBP-1's predictive value for RPL.

Results: The mean XBP-1 concentration in the RPL group was significantly higher than in the control group (p < 0.001). The mean values were 2243.65 ± 9425.27 pg/mL and 1196.32 ± 4378.81 pg/mL, respectively. The use of XBP-1 levels for the prediction of RPL was evaluated. In an ROC curve analysis, the area under the curve was found to be 87% (95% CI: 80% to 94.8%). The specificity was 78%, the sensitivity was 88%, the positive likelihood ratio (LR) was 4, the negative LR was 0.15, the positive predictive value was 80%, and the negative predictive value was 87% for the cut-off XBP-1 level at 1364.68 pg/mL.

Conclusion: This study highlights the potential role of ER stress in RPL and proposes XBP-1 as a predictive biomarker for pregnancy loss. Understanding ER stress mechanisms in RPL could inform diagnostic and therapeutic strategies. Further research is essential to validate these findings and explore their clinical implications.

## Introduction

Recurrent pregnancy loss (RPL) is conventionally characterized by three or more consecutive pregnancy losses, excluding ectopic and/or molar gestations, occurring before 20 weeks of gestation [1]. Under this definition, the reported prevalence of RPL ranges from 1% to 5% [2-4]. However, evolving demographics, particularly maternal age at first pregnancy, have prompted adjustments to the definition of RPL to prevent delays in couples' evaluations. As per the 2017 European Society of Human Reproduction and Embryology (ESHRE) guidelines, RPL is now defined as two or more pregnancy losses before 24 weeks of gestation [5].

The etiological data on RPL remains limited. A comprehensive definition of RPL encompasses the assessment of genetic, endocrine, anatomical, immunological, hematological, and environmental factors [6-10]. Nonetheless, these evaluations often yield no pathological findings, leading to cases categorized as RPL with unknown etiology. Given the higher likelihood of subsequent pregnancy success following aneuploid conceptus, experts advocate for cytogenetic analysis of products of conception over unnecessary and costly techniques like couple karyotyping [11].

The dynamic cellular, molecular, and genetic transformations occurring in both female and male reproductive tissues, such as oogenesis and spermatogenesis, commence in prenatal life. These processes necessitate extensive protein synthesis and maturation, which partly take place within the endoplasmic reticulum (ER) of cells. Additionally, the ER facilitates protein transport to appropriate cellular locations and oversees the degradation of unfolded or misfolded proteins. The accumulation of such proteins in the ER lumen leads to ER stress, triggering the unfolded protein response (UPR) to enhance ER-associated degradation and maintain ER homeostasis [12]. The X-box binding protein-1 (XBP-1), a transcription factor, plays a pivotal role in the UPR by activating specific genes and regulating ER stress-induced apoptosis [13].

Disturbed ER homeostasis contributes to the pathogenesis of various diseases, including diabetes mellitus, metabolic syndrome, non-alcoholic fatty liver disease, and hypertension [14-16]. Moreover, numerous studies highlight the significance of ER and UPR cascades in regulating various reproductive processes such as the menstrual cycle, ovarian folliculogenesis, oocyte maturation, spermatogenesis, fertilization, pre-implantation, embryo development, pregnancy, and parturition [12]. Furthermore, impaired ER homeostasis resulting from ER stress-mediated UPR signaling pathways contributes to several reproductive tissue pathologies, including endometriosis, cancer, and pregnancy complications associated with preterm birth [12].

However, evidence regarding the association between RPL and ER stress remains scarce. In this study, we aimed to assess ER stress in patients with RPL of unknown etiology by quantifying plasma concentrations of XBP-1 in RPL patients and comparing them with those of healthy subjects.

## Materials and methods

This prospective observational study was conducted at a tertiary obstetrics clinic in Istanbul, Turkey, from March 2020 to September 2020. The study protocol received approval from the Ethics Committee of the Bakırköy Sadi Konuk Training and Research Hospital, University of Health Sciences (approval no. 2020-83) and was registered with ClinicalTrials.gov (no. NCT04455256). Informed written consent was obtained from all participants prior to enrollment.

A total of 45 female patients aged 18 to 35 years, presenting at the gynecology outpatient clinic with a history of at least two unexplained pregnancy losses before 20 weeks of gestation during the study period, were enrolled in the study group. Previous pregnancies were confirmed by the presence of a gestational sac or fetus under a transvaginal ultrasound examination. Participants underwent comprehensive evaluations for infections, congenital uterine anomalies, karyotyping, genetic analysis for thrombophilia, and screening for conditions associated with an increased risk of pregnancy loss, such as antiphospholipid syndrome, uncontrolled diabetes mellitus, polycystic ovary syndrome, thyroid antibodies, adenomyosis, uterine leiomyomas, and hyperprolactinemia. Exclusion criteria comprised fetal anatomical abnormalities; infectious, endocrine, or genetic disorders; endometriosis; adenomyosis; uterine anomalies; hydrosalpinx; abnormal karyotypes; smoking; drug or alcohol abuse; and confirmed pregnancy. Additionally, patients with chronic or acute inflammatory or metabolic diseases, or those on steroids, anti-inflammatory, or antioxidant medications, were excluded. The control group consisted of 45 healthy women with a history of at least two previous uncomplicated pregnancies and no history of pregnancy loss or infertility.

Detailed medical histories were obtained, and all participants underwent physical and gynecological examinations. Body mass index (BMI) was calculated, and thyroid-stimulating hormone (TSH) levels were measured. Venous blood samples were collected during initial consultations, irrespective of menstrual cycle phase, between 9:00 and 11:00 in the morning to minimize circadian influences. Samples were centrifuged, and serum aliquots were stored at −80°C until analysis. Serum XBP-1 levels were quantified using the Human XBP-1 ELISA Kit (catalog no. E-EL-H557; Elabscience Biotechnology Inc., Houston, TX, USA), and optical density was measured at 450 nm using a standard automated plate reader (Thermo Fisher Scientific, Waltham, MA, USA). The kit's detection range was 78.13-50,000 pg/mL.

Data were analyzed using SPSS Statistics version 20.0 (IBM Corp., Armonk, NY, USA). Continuous variables are presented as the mean ± standard deviation. The distribution of clinical and laboratory variables was assessed using the one-sample Kolmogorov-Smirnov test. Parametric variables were compared using the Student's t-test, while non-parametric variables were assessed using the Mann-Whitney U-test. Categorical variables were compared using either Pearson's or chi-square tests. Sensitivity, specificity, positive predictive value (PPV), negative predictive value (NPV), and accuracy of XBP-1 were calculated. Receiver operating characteristic (ROC) curve analysis was conducted to determine a significant cutoff level of XBP-1 for predicting recurrent pregnancy loss (RPL). A p-value of <0.05 was considered statistically significant for all analyses.

## Results

A total of 45 patients in the RPL group and 45 patients in the control group were included in the study. The clinical data for the RPL and control groups are presented in Table 1. There were no significant differences between the groups in terms of age, BMI, and TSH levels, with p-values being 0.324, 0.942, and 0.642, respectively. The differences between the groups were observed in the obstetric history. The mean gravidity in the RPL group was 4.2±1.1, which was significantly higher than the control group (p<0.001), whereas the parity in the same group was significantly lower than the control group (p<0.001). The mean number of abortus among the RPL patients was 3.5 ± 0.7.

**Table 1:** Biometric data and the XBP-1 results RPL: Recurrent pregnancy loss, XBP-1: X-Box binding protein-1, BMI: Body mass index, TSH: Thyroid stimulating hormone

Parameters	Control group (n=45) mean±SD	RPL group (n=45) mean±SD	p-value
Age	25.57±3.58	26.44±3.76	0.324
Gravidity	1.3±0.4	4.2±1.1	<0.001
Parity	1.3±0.4	0.7±0.6	<0.001
Abortus	0	3.5±0.7	<0.001
BMI	21±2.48	20.9±2.53	0.942
TSH	1.81±0.67	1.75±0.73	0.642
XBP-1	1196.32± 4378.81	2243.65± 9425.27	<0.001

**Figure 1:**
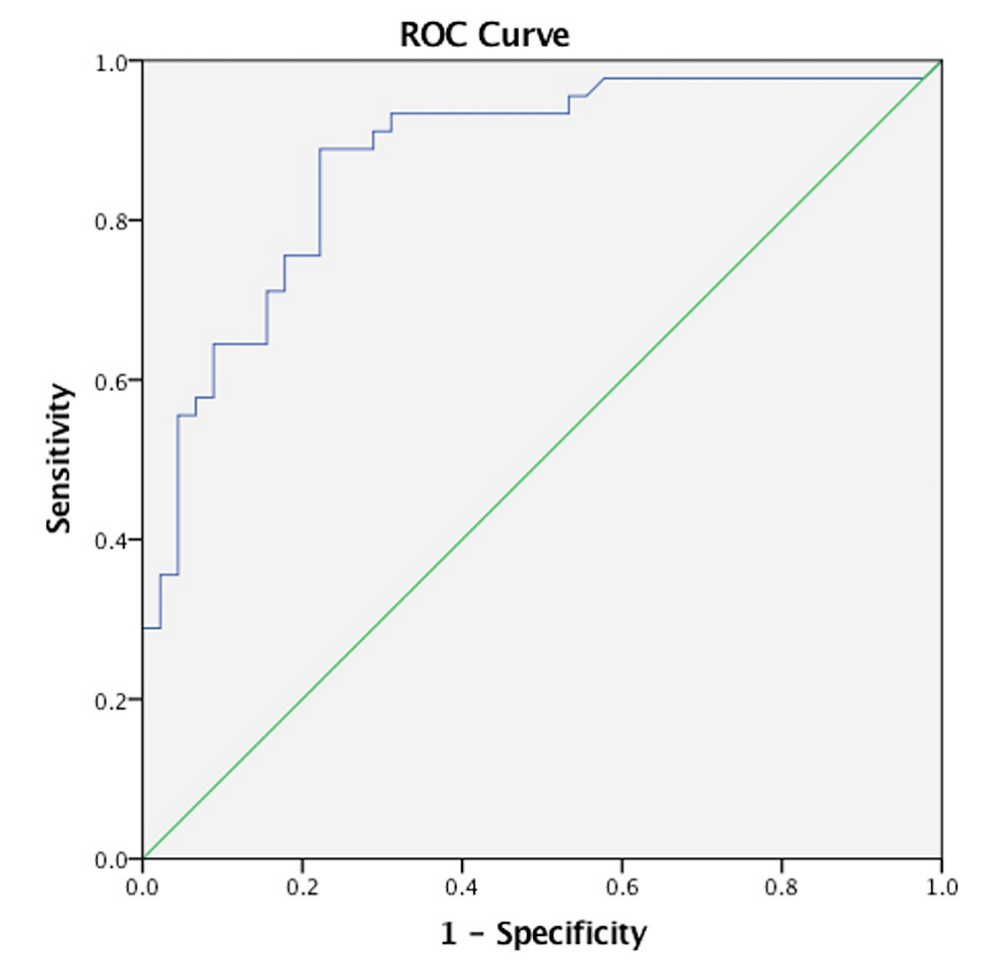
The ROC curve analysis of XBP-1 and RPL ROC: Receiver operating characteristic, XBP-1: X-box binding protein-1, RPL: Recurrent pregnancy loss

The mean XBP-1 concentration in the study group was significantly higher than in the control group (p < 0.001). The mean values were 2243.65 ± 9425.27 pg/mL and 1196.32 ± 4378.81 pg/mL, respectively. The use of XBP-1 levels for the prediction of RPL was evaluated. In the ROC curve analysis, the area under the curve was 87% (95% CI: 80% to 94.8%) (Figure 1). The specificity was 78%, the sensitivity was 88%, the positive likelihood ratio (LR) was 4, the negative LR was 0.15, the PPV was 80%, and the NPV was 87% for the cut-off XBP-1 level at 1364.68 pg/mL (Table 2).

**Table 2:** The ROC curve analysis of XBP-1 and RPL ROC: Receiver operating characteristic, XBP-1: X-box binding protein-1, RPL: Recurrent pregnancy loss, AUC: Area under the curve, PPV: Positive predictive value, NPV: Negative predictive value, LR+: Positive likelihood ratio, LR-: Negative likelihood ratio, CI: Confidence interval

XBP-1 cut-off value	AUC	p-value	Specificity	Sensitivity	PPV	NPV	LR+	LR-	95% CI	
									Lower	Upper
1364.68	87%	<0.001	78%	88%	80%	87%	4	0.15	80%	94.8%

## Discussion

This study aimed to elucidate the role of oxidative stress in RPL of unknown etiology by assessing serum XBP-1 levels in 45 RPL patients and comparing them with a control group comprising 45 healthy women. Elevated serum XBP-1 levels observed in the RPL group underscore the potential involvement of ER stress in RPL development. Additionally, XBP-1 levels may serve as predictive markers for pregnancy loss in individuals aspiring for fertility. To the best of our knowledge, this is the first investigation to explore the relationship between XBP-1 levels and RPL.

Recent studies examining early pregnancy loss, preeclampsia, and pregnancy complications such as hydatidiform mole propose a common pathophysiological oxidative stress pathway among these conditions [17-19]. Evidence suggests that ER stress, mediated by UPR activation, contributes to abnormal placentation in early pregnancy, while ER stress-related endothelial dysfunction is implicated in the onset of pregnancy complications such as gestational diabetes mellitus, obstetric cholestasis, and preeclampsia [20-23]. However, the literature regarding biomarkers for predicting RPL remains sparse.

The ER plays a pivotal role from spermatogenesis to embryo formation [24,25]. Studies indicate that testicular hyperthermia induces UPR signaling cascades, potentially impairing spermatogenesis [26]. Chow et al. reported an association between elevated XBP-1 levels and male infertility [27]. Similarly, the ER exerts significant influence during oocyte production, with regulation of ER homeostasis and stress emerging as critical mechanisms during folliculogenesis, oocyte maturation, and embryogenesis. Elevated XBP-1 levels in cumulus cells from fertilized oocytes suggest the physiological involvement of UPR signaling in oogenesis and fertilization [28]. Severe ER stress impedes blastocyst formation via extensive apoptosis, resulting in abnormal embryonic development [25]. The ER stress induced by fatty acids also disrupts protein secretion and mitochondrial activity, culminating in aberrant embryonic development. Animal models further demonstrate that ER stress induces DNA damage, contributing to fetal anomalies during the early gestational weeks [28].

Successful embryo implantation necessitates a stable microenvironment regulated by a delicate balance between immune and inflammatory responses. Excessive cytokine levels can trigger calcium release from the ER, leading to reactive oxygen species generation, ER stress, and inflammation, thus perturbing the uterine microenvironment [29]. The elevated XBP-1 levels observed in the RPL group compared to controls could potentially stem from one of these factors, ultimately leading to increased ER stress.

Limitations

This study includes a small sample size and relies solely on serum samples for determining XBP-1 levels. Blood samples were collected irrespective of patients' menstrual cycles due to a lack of data suggesting menstrual cycle effects on ER stress. Furthermore, standardization of the temporal interval between the last abortion in the RPL group and the last birth in the control group was not feasible due to the observational study design. Future studies should consider standardizing sample collection procedures and integrating aneuploidy testing of abortus material. Validation of these findings warrants multicenter studies with larger sample sizes. Nevertheless, this study's pioneering evaluation of ER stress in RPL using XBP-1 levels represents a notable strength.

## Conclusions

The investigation explored the role of oxidative stress in RPL of unknown etiology by assessing serum XBP-1 levels in patients with RPL compared to a control group of healthy women. Elevated serum XBP-1 levels observed in the RPL group suggest the potential involvement of ER stress in the development of RPL. Furthermore, XBP-1 levels may serve as predictive markers for pregnancy loss in individuals aspiring for fertility. This study contributes to the understanding of the pathophysiological mechanisms underlying RPL and highlights the potential utility of XBP-1 as a biomarker in RPL diagnosis and management. Future research with larger sample sizes and multicenter studies is warranted to validate these findings and explore the clinical implications further.
